# Academy of Stomatology

**Published:** 1902-11

**Authors:** 

**Affiliations:** Academy of Stomatology


### ACADEMY OF STOMATOLOGY.

A REGULAR meeting of the Academy of Stomatology of Philadelphia was held at its rooms, 1731 Chestnut Street, on the evening of Tuesday, March 25, 1902, the President, Dr. S. B. Luckie, in the chair.

A paper entitled "The Value of Cataphoresis" was read by Dr. Louis Jack.

(For Dr. Jack's paper, see page 781.)

### DISCUSSION.

*Dr. J. A. Woodward.*—I have listened with a great deal of pleasure to Dr. Jack's paper. He has covered the subject thoroughly from a practical stand-point. I commenced the use of cataphoresis in May, 1896, and have made, up to the present time, three hundred and forty-six applications at an average of about fifteen and three-eighths minutes for the actual passage of the current. A little more time is required in placing the rubber dam and in fixing the electrode in place. In cases where a local anæsthetic is required, I have nothing which will produce so deep an impression upon the dentine as cocaine cataphorically applied. The patients requiring it are usually those who are unable or unwilling to bear much pain. Sometimes it is a delicate woman, at other times a child; or, again, an older person. I do not think I have ever failed to produce such an impression on the dentine as will reduce its sensibility to some degree and enable the patient to bear the operation with reasonable comfort. If more time be taken, one can nearly always produce entire insensibility. Success seems to depend almost entirely upon confining the current directly to the cavity.

There is a different degree of response to the electrical current in different people. In the teeth of the same individual there is not much difference, but in the mouth of different individuals there is a great difference, and that is probably the reason why many men have failed with cataphoresis. Where a small amount of current gives pain they do not persist long enough, and, of course, they do not get the desired effect. Some of these extreme cases take forty minutes of current application. As to the possible damage to tooth and pulp, I have never seen it to any degree. Nirvanin seems to be quite as effective as cocaine.

*Dr. E. T. Darby.*—I have been very much interested in Dr. Jack's paper, and know that any statement that Dr. Jack makes is thoroughly reliable as well as interesting. I have had very little—I may say, no experience in my office with cataphoresis, but I have known of others who have had such satisfactory experiences that I am tempted to relate one case that came under my observation a year ago. A lady had in her mouth at least thirty cavities to be filled. She impressed me as being of a supersensitive organization, and I concluded that if she became my patient I would have quite a siege with her, because many of the operations were large. She was about to be married, and asked to have all the work done within a month. It being impossible for me to do this, her *fiancé* suggested that she employ his dentist at New York. This was done, and during a period of thirty days she sat two hundred and three hours in the dentist's chair. I said to her mother that I expected to find her pretty well used up. She came back and showed me what had been done. Her mouth had been most beautifully attended to; every operation had been made with gold, and many of them were very large indeed.

I said to her, "I am surprised to find that you look so fresh after so many sittings within a month," but she replied that the dentist had used cataphoresis throughout the work, and that she had suffered no pain whatever.

The teeth were not in the slightest degree hypersensitive to thermal changes after the operations.

I then began to think that cataphoresis was a very good thing. I have seen two patients since then who have been in the hands of this gentleman, and they report the same result. Now, this testimony, coming from the patients, ought to be reliable, for they have no reason to state other than the truth. My own opinion of cataphoresis has been that too much time is required to produce the desired effect, so that I have not felt able to give it to my patients. Possibly I have been a little cruel in causing pain which I might have avoided by applying cataphoresis. I have found cases in which I have obtunded sensibility by using Robinson's remedy, or alcohol and hot air, and recently I have used boiling carbolic acid, and cocaine on cotton, as recently suggested by Dr. Jenkins. The first contact of the carbolic acid to the cavity causes a little pain, but it is immediately followed by a soothing effect, and in these cavities where there is a large amount of decomposed dentine I think the result is instantaneous. In the removal of that soft, leathery decay from the deeper portions of the cavity I use Robinson's remedy, followed by alcohol and blasts of warm air, and I have very little complaint on the part of my patients when it is necessary to do any cutting. As I said before, in any statement that Dr. Jack has made I would have the most absolute faith.

*Dr. E. C. Kirk.*—I am very much interested in the subject of Dr. Jack's paper, but my experience has been quite limited in the use of cataphoresis for obtunding sensitive dentine. The most frequent use that I have made of it has been in the treatment of pulpless teeth for removal of discoloration. I would like to ask a question which Dr. Jack may be able to answer for me. Is there a point at which he would limit the application of cocaine by the cataphoric method, with a view to avoid disturbance of the pulp by the electric current itself; in other words, the danger of the electrocution of the pulp, as it has been called? It seems to me that in careless hands the method might possibly result occasionally in destruction or impairment of the function of the pulp. In fact, the case reported by Dr. Darby, in which such extensive gold operations were performed, there being no after-hypersensitiveness, sounds a little ominous as to the condition of these pulps. I would like to know if in the technic employed in the treatment of cases of this kind there is any means of determining when enough has been done and not too much; if there is any variation in the resistance of the tooth during the process of anæsthesia, which would indicate a possible deleterious effect upon the pulp.

*Dr. Jack.*—It would probably be better to answer this question as well as I may at the moment it is propounded. I may say to

Dr. Kirk that in my first experience I was guided more by the time than by my instrument, and I would often stop after fifteen minutes, although I may state here that the shortest application which produced complete anæsthesia lasted five minutes. There is a class of persons whose teeth are much irritated by a feeble current. It is with these we find the highest degree of sensitivity and the greatest need for this mode of treatment. With these electrical irritation sets in at about four hundred thousand ohms' resistance, or when, as measured by the ampèremeter, one-twentieth of a milliampère of current is passing through the tooth. When the instrument shows one-tenth, there is no difficulty, and the operation will go on with considerable facility; but where the first show is only one-twentieth or one-fortieth of a milliampère, the time will be longer. I move the controller to a point of slight irritation; then go back one pin, and the irritation ceases. I then move it along a few pins at a time. On the appearance of the pain limit I go back. I now usually go clear around the circuit until all resistance is removed, and in that way I find complete anæsthesia. I have never found any after-disturbance, and think there is comparatively little danger of disturbing the pulp. It is more difficult, in many instances, to anæsthetize a pulp than to anæsthetize the most excessive sensitivity of the dentine. In some instances I have been able to anæsthetize a pulp and remove it in fifteen minutes, but in other cases a longer time is required.

*Dr. Kirk.*—Did you notice any difference in the rate of current flow, or a difference of amperage, as the dentine became saturated with cocaine? I mean, do you notice that the amperage changes when the controller-arm is placed at a definite point and a certain amperage is at first recorded?

*Dr. Jack.*—The amperage remains constant until the voltage can be increased by lessening the resistance. It will be found often that at first we can advance only very slowly because of the electrical irritation, but as we get towards the half-distance we advance more quickly.

*Dr. Darby.*—How about subsequent sensitivity to heat and cold?

*Dr. Jack.*—If the cavity was not carbolized, there would be high sensitivity to cold.

*Dr. Darby.*—How do you account for the case I mentioned in which there was no sensitivity after treatment?

*Dr. Jack.*—Probably carbolic acid, which coagulates the albumin of the fibrillæ, was used to prevent after-response to thermal change.

*Dr. H. E. Roberts.*—The time required depends upon the amount of cocaine introduced, and that in turn upon the amount of current passing through the tooth. Naturally more cocaine is introduced with a one-tenth milliampère than with one-twentieth, hence at the latter degree of flow twice the length of time would be consumed to attain a result.

I have never seen the amperage change at a given point on the controller. The lessened resistance from the increased amount of cocaine in the dentine would be so infinitesimal that it would be impossible to measure it when taken in conjunction with the resistance of the body added to the resistance of four hundred thousand ohms. If the electrode is not held perfectly immovable, you can get a variation in the milliamperage. I have not used cataphoresis of late, principally upon the ground that it takes so much time; but at one time I experimented with it thoroughly, and I believe that I have never had a failure to obtund the sensibility of the dentine with it, provided I could apply the rubber dam and keep the cavity dry. The patient has to be susceptible to the influence of cocaine. I have been compelled at times to stop the application simply because of the nervousness of the patient. In one case I thought the patient was going to faint, but she did not. One of the objections I held was the difficulty of getting the patient to understand that there was nothing to be feared. When I had an intelligent patient who understood it, I got along satisfactorily. I have used cataphoresis in bleaching, but have not done so for several years, as I can get equally good results without it. I have also used cataphoresis in obtunding a sensitive gum when I desired to make an artificial fistula. The burning from the direct current seems to me different from the action of any other cautery. By wrapping a plug of cotton around the electrode point, saturating that with cocaine, pressing gently against the gum, and gradually putting on the current, you can cauterize the gum, so that it will appear very much like boiled meat. There will be a depression which you can easily go through. The non-sensitivity does not extend to any great distance from the cauterized portion. I have had blisters on the back of the hand from cataphoresis and the scars did not disappear for months. For that reason I would apply the cathode only to the palm of the hand, where the skin is better able to resist burning; also, the sponge should be kept well wet. I have never seen pulps devitalized by the use of cataphoresis.

*Dr. Woodward.*—To prevent leakage of the current it is safer to apply the rubber dam only to the tooth to which the application is to be made. The electrode should cover as much of the floor of the cavity as possible, for the reason that a large electrode anæsthetizes a larger area of dentine than a smaller one. Cotton which has been saturated with the cocaine solution should be tightly packed around and between the electrode and the dentine to prevent contact or any movement.

The current is increased as rapidly as the pain limit will permit, until one-tenth milliampère is recorded. I usually wait at this amperage for five minutes, during which time there is not any change in the reading of the milliampèremeter. A large amount of current is not required. One-tenth to two-tenths of a milliampère will relieve or reduce sensitiveness sufficiently if one will have patience. With a meter the amount of current is shown; this, with the time of application and the loss of response of the tooth to the current, will afford a guide as to the point at which to limit the application. As to blistering any patient's hand, I cannot conceive how that could be done in ordinary cataphoric work.

*Dr. Roberts.*—That happened from a cataphoric application for bleaching, when I placed several layers of blotting-paper on the back of the hand and the cathode upon them. I was bleaching three teeth at one time, and the current was turned on for nearly two hours. The patient did not complain, but merely said that the hand felt a little warm. That was the only indication given.

*Dr. Jack.*—In this connection I might state that my cathode has always been placed on the cheek, unless the person has too much adipose tissue, when I would place it in the hand.

*Dr. Roberts.*—You are using much less current than in bleaching. I was using thirty volts.

*Dr. William Trueman.*—It seems to me that the question propounded by Dr. Kirk is a very important one in this connection. The question has been asked repeatedly in our journals, "Who to-day uses cataphoresis?" Now, there is a reason for this, and, as I understand it, that is that pulps died under the operation. Whether this was due to the fact that the men who used the current with cocaine did not understand it, or whether they carried it too far, the fact is well understood that pulps have died.

It is important to know when the paralysis that is effected by cocaine and the current should be stopped. The sensory nerves become paralyzed, and if you carry that to a great degree you stop the nutrition of the pulp, and it must die. That is the theory, and I believe it to be correct. If this association, through Dr. Jack, Dr. Woodward, and those who use it, can remove the objections of the profession to cataphoresis, it will be a good thing. I have not only great faith in Drs. Jack and Woodward, but in cataphoresis itself. I do, however, want to know, as Dr. Kirk does, how far to carry it in order to obtain good results and avoid the ill effects of it.

*Dr. Jack.*—In my experience I have not known of a single instance in which a pulp has been disturbed or died as a result of the application. I think that much of the good result of the work of myself and Dr. Woodward is due to the properties of the apparatus we use. It is capable of better control than any other one with which I am acquainted. There are two points bearing on the subject that will meet Dr. Trueman's objections, and which indicate that there is not much liability to the disturbance of the pulp. I have found repeatedly that sensitivity would recur within five minutes after I prepared the cavity. In some instances, when I prepared a cavity on one day and the patient came to me on the next, I found all parts of the margins of the cavity extremely sensitive, notwithstanding the fact that immediately after the preparation of the cavity it was dressed with carbolic acid. That would indicate that the pulps have not been disturbed. It proves that the pulp, in a large majority of these cases, is not the structure impressed by the cocaine. It is the dentine itself, because, in quite a number of instances, while we may prepare the cervical and lateral walls of a proximal cavity, it will be found at the occlusal margin that there will be some sensibility, showing that the current seeks the lines of least resistance, and by going in a direct course has failed to act upon the occlusal margin of the cavity. The impression is not made primarily on the pulp, unless it happens to be very nearly exposed, but even if it is I have not found disturbance. I can understand how persons at the beginning of the application, with even a good controller, may by suddenly throwing on quite a large amount of current produce injury of the pulp. At a given point the amperage remains the same without any elevation. I may state further that at the first point of electrical irritation the amperage will be the same with ten, fifteen, twenty, or twenty-five cells. The only difference is that the resistance must be greater as we increase the number of cells, but the amperage will be the same, although it must be stated that these ampère meters are not exactly fine; they are not capable of registering the most delicate moves. I use twenty-four per cent. cocaine, sometimes only twelve per cent. My method of using cocaine is to have powders containing one and one-fifth grains. The controller has one hundred and thirteen stops, or fractional volt selections, and a total voltage of less than twenty-four volts. I usually use fifteen cells, or about thirteen volts.

*Dr. Woodward.*—I have filled some of these cataphorically treated cavities with gutta-percha, and have removed it within twenty-four hours, six months, or three years. I find the teeth apparently as sound as any tooth, as far as sensitivity and response to temperature changes are concerned, and many times would not know that the tooth had been treated cataphorically until I referred to my record. I have frequently made an application to a cavity in one surface of a tooth, relieving the sensitiveness entirely, and found that a cavity in another surface remained as sensitive as if no cocaine had been applied. If the pulp had been anæsthetized, this would not be.

Otto E. Inglis, D.D.S.
*Editor Academy of Stomatology.*

